# Value of couch height-based positioning in postoperative adjuvant radiotherapy for left-sided breast cancer

**DOI:** 10.1038/s41598-025-24591-7

**Published:** 2025-11-19

**Authors:** Zaichun Shang, Jian Chen, Ming Li, Binbin Ge, Jinjin Feng, Jianhua Jin, Jianting Wu, Hanzhen Ji, Kaiyue Chu, Xinhua Zhang

**Affiliations:** 1 Department of Radiotherapy, Nantong Tumor Hospital, The Afffliated Tumor Hospital of Nantong University, Nantong, China; 2 Department of Radiotherapy, Affiliated Hospital of Nantong University, Nantong, China; 3 Department of Anatomy, Medical School of Nantong University, Nantong, China; 4 Department of Hospital Library, Nantong Third Peoples Hospital, Nantong, China; 5 School of Electronic Science and Engineering, Nanjing University, Nanjing, China

**Keywords:** Breast cancer radiotherapy, Positioning error, Treatment couch height, Dose distribution, Outcomes research, Breast cancer, Breast cancer, Cancer therapy, Biological techniques, Biophysics, Cancer

## Abstract

This study aimed to evaluate the value of positioning based on the treatment couch height in radiotherapy for left-sided breast cancer. Sixty patients who had undergone radical mastectomy for left-sided breast cancer were selected, with each patient undergoing positioning based on the treatment couch height (couch height group) and positioning based on the reference skin marking lines (reference line group), to measure corresponding positioning errors. Meanwhile, 20 of 60 patients were randomly selected, and the planning system was used to simulate the changes in radiation doses in planning target volume (PTV) and organs at risk (OARs) along with the changes in positioning errors in dorsal (increasing couch height) and ventral (decreasing couch height), respectively. Compared with the original plan, when the positioning error in the dorsal direction reached 3 mm, D_mean_, V_30 ,_ and V_20_ in the ipsilateral lung were increased by 35.12%, 16.35%, and 10.6% respectively, and V_50_ in PTV was decreased by 0.99% (all *p* < 0.05); when the positioning error in the ventral direction reached 1.5 mm, V_50_, V_48_, and V_45_ were decreased by 2.07%, 0.58%, and 0.14% respectively. The homogeneity index (HI) was increased by 14.28% (all *p* < 0.05). There was a statistically significant difference in the positioning errors in the ventral-dorsal directions between the couch height group (0.16 ± 0.14 cm) and reference line group (0.36 ± 0.25 cm) (*p* < 0.05); the percentages of the absolute positioning errors within 1.5 mm and 3 mm were 52.4%, 88.7% respectively in the couch height group and 29.8%, 54.4% respectively in the reference line group, (all *p* < 0.05). Dorsal positioning errors greater than 3 mm significantly worsen the dose distribution for both the PTV and OAR, while positioning based on the treatment couch height keeps 88.7% of positioning errors within 3 mm; ventral positioning errors greater than 1.5 mm result in significant changes in the dose within the PTV. Compared to the reference line group, positioning based on the treatment couch height controls 52.4% of positioning errors within 1.5 mm. Therefore, couch height-based positioning demonstrates greater advantages in managing ventral-dorsal positioning errors. This study provides a reference for clinical positioning in postoperative adjuvant radiotherapy of breast cancer.

## Introduction

 The incidence of breast cancer is increasing year by year, which has seriously threatened the physical and mental health of women^1^. Radiotherapy, as an important adjuvant therapy after breast cancer surgery, has greatly improved the treatment outcome of patients^2^. The principles of radiotherapy are to ensure that the planning target volume (PTV) receives the prescription dose of radiation while the surrounding normal tissues receive a dose of radiation as little as possible. The study of Van Mourik A. et al. on the effects of positioning errors and breast-shape changes on target dose distribution showed that systematic errors of more than 1–2 mm and random errors of more than 2–3 mm would result in a dose loss of about 6% in the proximal dorsal region and about 2% in the skin margin^3^. Controlling the positioning errors in radiotherapy is an important part of radiotherapy quality control.

When the patients with left-sided breast cancer undergo radiotherapy, the heart is inevitably exposed to radiation, thus increasing the risk of ischemic heart disease. This risk is directly proportional to the mean heart dose (MHD)^4^. Deep inspiration breath-hold (DIBH) technique can effectively reduce irradiated doses in the heart and lungs. This technique involves asking the patients to hold their breath after taking a deep breath, using lung expansion to push the heart away from the chest wall, thereby significantly increasing the distance between the heart and the radiation field, ultimately substantially reducing the irradiated dose in the heart, particularly the left anterior descending coronary artery^5^. According to study by Park S. et al.^6^, compared to free breathing, DIBH reduces the average cardiac radiation dose from 8.4 ± 1.3 Gy to 2.0 ± 1.1 Gy and the average lung radiation dose from 7.8 ± 1.5 Gy to 3.7 ± 1.4 Gy. The study further indicated that in the DIBH state, the dorsal positioning error has the most significant impact on organs at risk (OARs): when the positioning error reaches 5 mm, the average irradiated doses in the heart and lungs will deviate by 49.4% and 26.1% respectively.

At present, although a study suggested that surface guided radiotherapy (SGRT) can be used to replace skin marking line-based positioning^7^, this conclusion still needs to be verified by large-sample multi-center studies, and the equipment cost is relatively high, which restricts its popularization in grassroots hospitals. Skin marking line-based positioning remains the mainstream technique in radiotherapy centers^8^, but it is easily affected by factors such as skin laxity, weight changes, and blurred marking lines, and positioning errors control remains a challenge in clinical practice. The meta-analysis by Costin I. C. et al. indicated that position-stabilizing devices can help to reduce positioning errors^9^. The study by Gao Han et al. demonstrated that the breast bracket combined with vacuum pad fixation outperforms the vacuum pad fixation alone^10^. Nevertheless, even with these methods, our institution has observed significant variability in ventral-dorsal positioning errors.

As early as 1998, Greer P. B. et al. first proposed the use of couch height-based positioning as a reference standard for controlling ventral-dorsal positioning errors in pelvic tumors^11^. The conclusion was subsequently validated by multiple studies and gradually applied in radiotherapy positioning for abdominal^12^ and nasopharyngeal^13^ tumors. In 2023, ESTRO ACROP consensus guideline for positioning, immobilization and setup verification for local and loco-regional photon breast cancer irradiation recommended couch height-based positioning, in which the vertical heights of the treatment couch (with corresponding couch height readouts on the linear accelerator) are used as a stable and reproducible geometric reference coordinate. Compared with traditional skin marking line-based positioning method (e.g., laser alignment based on skin marking), this approach reduces ventral-dorsal positioning errors, thus minimizing dose deviations and possibly obtaining better dosimetric results^14^.

Based on prior studies^11–13^, this study analyzes the variation patterns of radiation dose distributions in PTV and OARs with ventral-dorsal positioning errors to determine the error threshold in this direction, compares the magnitude and distribution characteristics of errors between two positioning methods such as couch height-based positioning and reference line-based positioning, evaluates their reference values in positioning for left-sided breast cancer radiotherapy, thereby providing evidence for clinical practice.

## Materials and methods

### General clinical data

A total of 60 patients who were treated in The Affiliated Tumor Hospital of Nantong University from 2022 to 2023 were recruited into this study. Inclusion criteria: (1) patients with pathologically confirmed left-sided breast cancer after radical mastectomy. (2) those without undergoing postoperative breast reconstruction; (3) those needing to receive adjuvant radiotherapy after surgery. Exclusion criteria: (1) male breast cancer patients; (2) those undergoing breast-conserving surgery; (3) those with severe underlying diseases who cannot successfully complete radiotherapy; (4) those with incomplete clinical data. The median age of the enrolled patients was 50 years (range: 25–78), and the median body mass index (BMI) was 24.56 Kg/m^2^ (range: 18.41–28.90). This study was conducted in accordance with the Declaration of Helsinki and approved by the Medical Ehics Committee of Nantong Tumor Hospital, The Affiliated Tumor Hospital of Nantong University (Approval No.2021-092). Written informed consent was obtained from each participant prior to study initiation.

### Materials

Computed Tomography (CT) (BrillianceTM, Philips, Amsterdam, Holland), Treatment Planning System (TPS) (Pinnacle3 9.0, Philips, Holland), Treatment machine (Elekta Synergy, UK), and Position fixing device (Coleridi Medical Equipment Co. LTD, Guangzhou, China).

### CT simulated positioning and plan design

The scanning range was from the cricothyroid membrane to 6 cm below the breast fold, CT scanning parameters were as follows: tube voltage 120 kv, tube current 400 ms, and layer thickness 5 mm. A water-equivalent bolus was placed over the skin corresponding to the treatment field in planning CT and throughout all treatment sessions to increase the irradiated dose in the skin surface of the chest wall and reduce the risk of local recurrence^15^. The scanned CT images were uploaded to the radiotherapy TPS, and the doctors delineated the clinical target volume (CTV) and OARs according to the ESTRO ACROP consensus guideline^16^. The CTV included ipsilateral chest wall, supraclavicular lymph, and axilla-level lymph. Based on previous studies conducted by our center and relevant literature^17,18^, considering possible positioning errors and internal organ motion, a uniform CTV-to-PTV margin of 0.5 cm was applied in all directions, and this margin was defined according to our institutional protocol. The treatment plan was optimized with the following criteria: at least 95% of the PTV volume received 50 Gy and 95% of the prescription dose covered at least 99% of the PTV volume; the hot spot was defined as a PTV receiving more than 110% of the prescription dose as little as possible; the radiation field was set to seven areas of irradiation at the angles of 300°, 330°, 0°, 30°, 60°, 90°, and 120°^19^. The primary breast tangent field was supplemented with a vertical chest wall field to improve lymphatic coverage without significantly increasing dose in the heart or lungs. To account for respiratory motion, a 2 cm external auxiliary structure was added to minimize target miss, and the MLC was appropriately opened during optimization. The prescription dose was 50 Gy in 25 fractions. The OARs included heart, ipsilateral lung, contralateral lung, healthy breast, humeral head, and spinal cord. The dose limits for OARs were as follows: ipsilateral lung: D_mean_ <15 Gy, V_30_ < 20%, V_20_ < 28%, and V_10_<55%; heart: D_mean_ < 8 Gy, V_30_<15%, V_10_<25%, and V_5_ < 40%; healthy breast: D_mean_ < 5 Gy; contralateral lung: V_5_ < 20%; ipsilateral humeral head D_mean_ < 30 Gy; spinal cord D_max_ < 45 Gy.

**Fig. 1:**
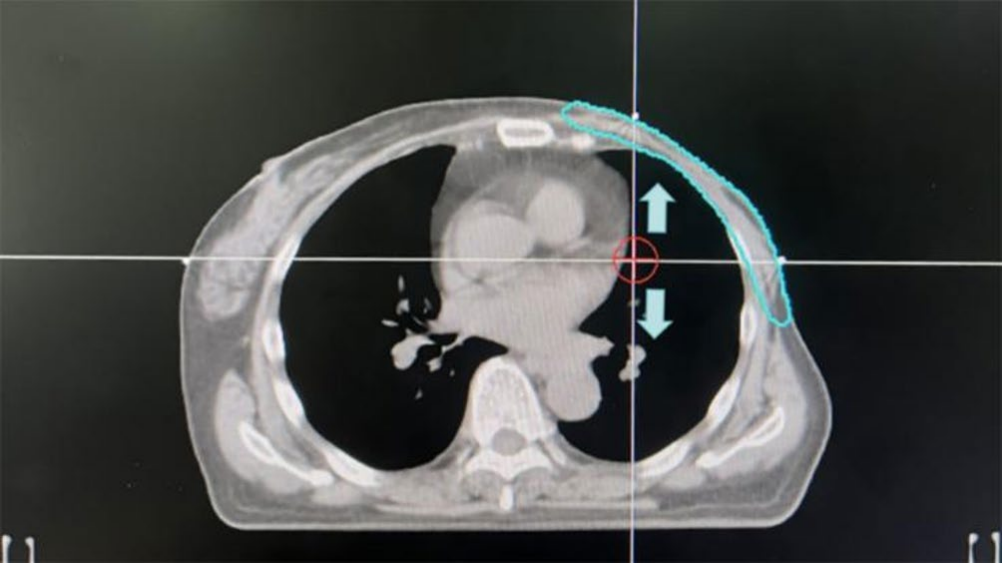
Schematic diagram of isocenter in cross-section of left-sided breast cancer. The area within the blue line was PTV. The isocenter was moved once every 1.5 mm along the arrow direction to simulate the positioning errors in the ventral-dorsal directions until corresponding positioning errors reached 9 mm; other parameters remained unchanged, and the planning system was used to calculate the radiation dose distribution in PTV and OARs according to different isocenter positions.

When all the parameter conditions of the original treatment plan remained unchanged, the isocenter was vertically moved once every 1.5 mm in the ventral (decreasing couch height) and dorsal (increasing couch height) directions of the patients to simulate the positioning errors in the ventral-dorsal directions until the corresponding positioning errors reached 9 mm. A total of 12 simulated isocenters were obtained for each patient. The planning system could calculate radiation dose distribution in PTV and OARs at different isocenters, respectively (Fig. 1).

A total of 240 simulated plans were obtained from 20 patients. The data were collected using TPS, including the percentages of PTV (V_50_, V_48_, V_45_) covered by the radiation doses (50 Gy, 48 Gy, 45 Gy), approximate maximum irradiated dose (D_2%_), approximate minimum irradiated dose (D_98%_), mean irradiated dose (D_mean_), homogeneity index (HI), OAR V_30_, V_20_ and V_10_, and V_5_ represented the percentages of irradiated volumes in OARs at a dose of 30 Gy, 20 Gy, 10 Gy, and 5 Gy, respectively.

The HI value was shown in Formula 1, D_2%_, D_98%_, and D_50%_ were the irradiated doses in different percentages (2%, 50%, 98%) of PTV. The smaller the HI value, the better the homogeneity of dose distribution in the PTV.

1 $$\:\text{HI}\text{=}\frac{{\text{D}}_{\text{2}\text{\%}}\text{-}{\text{D}}_{\text{98}\text{\%}}}{{\text{D}}_{\text{50}\text{\%}}}\text{}$$

### Treatment positioning and position verification

**Fig. 2:**
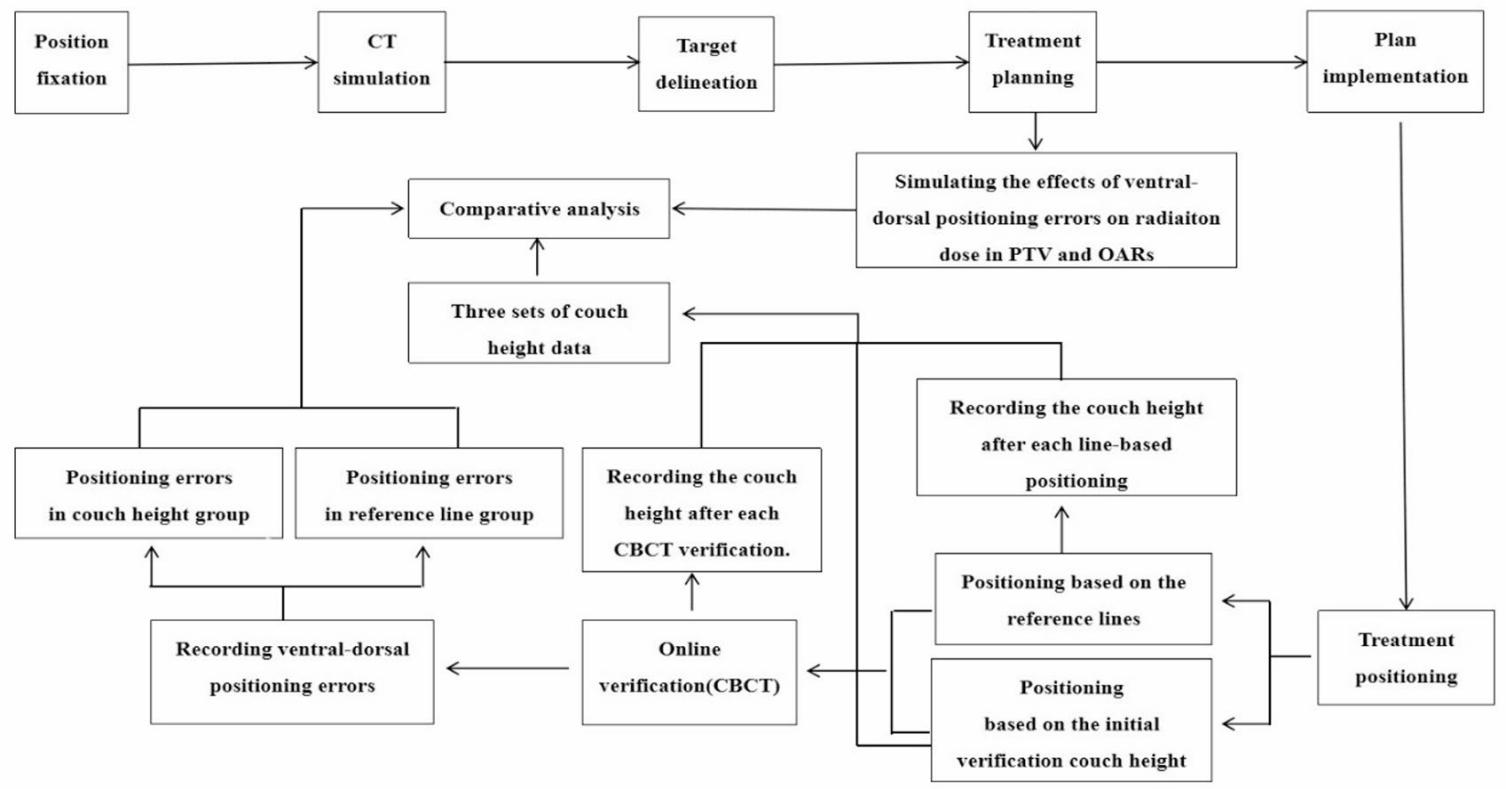
Flow chart of study design.

All patients were immobilized with breast brackets combined with vacuum pads. The first positioning of the patient was done with the joint participation of a radiology doctor, a physicist, and two radiotherapists. During the first treatment, the radiotherapists positioned the patient based on the skin marking lines on the patient’s surface, aligning the skin marking lines with the laser lines in three dimensions. Following patient positioning, online verification was performed using cone-beam CT (CBCT). All acquired scan images were registered to the planning CT using an automatic grayscale-based algorithm followed by manual fine-tuning, and all operations were performed by the same supervising radiotherapist. Three-dimensional positioning errors were calculated based on these registrated images. Thereafter, the treatment couch was automatically adjusted to correct the identified positioning errors. During the second treatment, the radiotherapists positioned the patient based on the skin marking lines on the patient’s surface and then raised the treatment couch to the initial verification height for online position verification. The positioning for the third treatment was the same as the first, while the fourth treatment follows the second, and so on. The positioning errors in both the reference line group and the couch height group were detected and recorded, respectively. CBCT was performed once daily. The 60 patients were expected to complete 1500 CBCT verifications; however, due to machine malfunctions and other factors, only 1276 verifications were completed—638 in the couch height group and 638 in the reference line group (Fig. 2).

### Couch height data acquisition

After each online verification, every patient had three sets of couch height data: Reference couch height (RCH), reference line-based couch height (RLCH), and verified couch height (VCH). RCH was the couch height after the initial verification and served as the reference couch height for subsequent positioning. RLCH was the couch height after positioning based on the skin marking lines. VCH was the couch height after each CBCT verification.

### Statistical methods

IBM SPSS statistics 26.0 was used for data statistical analysis. The data were plotted in GraphPad Prism 9.4.1. The pared-samples T-test was performed to compare the differences in positioning errors in the ventral-dorsal directions between the couch height group and the reference line group; the chi-square test was used to compare the constituent ratios. The independent samples *t*-test was performed to compare the differences in radiation dose distribution in PTV and OARs between the original and simulation plans. The results were expressed as mean ± standard deviation (M ± SD), *p* < 0.05, indicating a significant difference. Pearson correlation analysis was conducted to explore the relationships of RCH and RLCH with VCH.

### Estimation of the margin of planning target volume

To account for interfractional variations, PTV was expanded from CTV in all directions, and the margin of planning target volume (MPTV) was calculated using the following Van Herk formula^20^: MPTV = 2.5Σ + 0.7σ, where Σ represents the standard deviation of a given systematic errors, which is defined as the standard deviation of the individual mean positioning errors across the population, and σ denotes the standard deviation of random positioning errors, which is computed as the root mean square of the standard deviations of the daily positioning errors for each patient.

## Results

### Evaluation of various PTV indicators

**Fig. 3:**
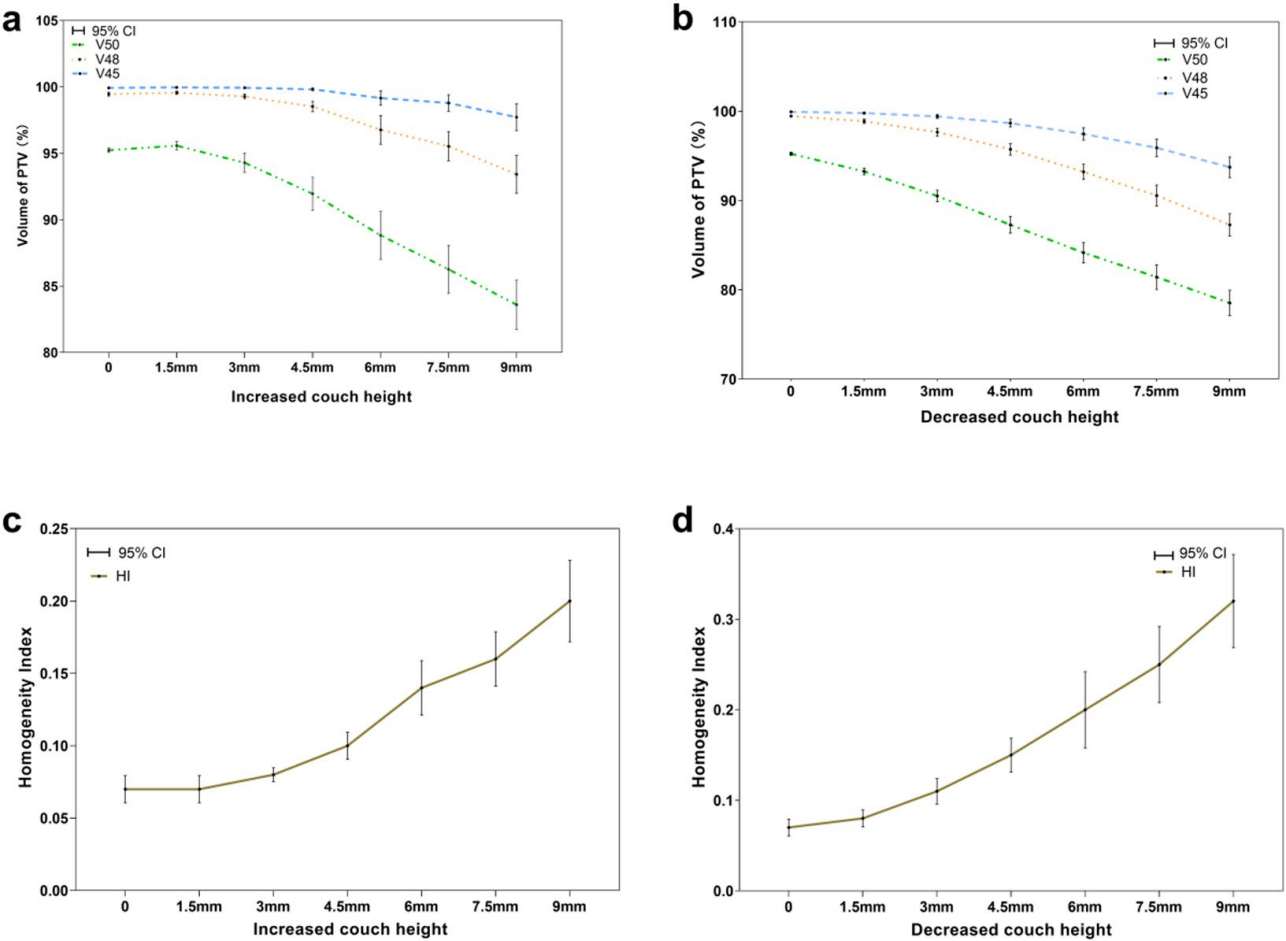
Effect of couch height changes on the radiation dose distribution in PTV. (**a**, **b**) Effects of increasing and decreasing couch heights on radiation dose distribution in PTV. (**c**, **d**) Effects of increasing and decreasing couch heights on HI.

Compared with the original plan, PTV covered by an equivalent dose showed an overall decreasing trend along with the changes in treatment couch height, while HI showed an overall increasing trend along with the changes in treatment couch height (Fig. 3). When the couch height was increased by 3 mm, V_50_ was decreased by 0.99%, HI was increased by 14.29% (both *p* < 0.05); When the couch height was decreased by 1.5 mm, V_50_, V_48_, and V_45_ were decreased by 2.07%, 0.58%, and 0.14% respectively, and HI was increased by 14.28%. when the couch height was decreased by 3 mm, V_50_, V_48_, and V_45_ were decreased by 4.96%, 1.8%, and 0.51%, and HI was increased by 57.14%, respectively (both *p* < 0.05) (Table 1).

**Table 1:** Effects of changes in couch height on the radiation dose in PTV.

*N* = 20	V_50_ (%)	V_48_ (%)	V_45_ (%)	HI (%)
Increased couch height (mm)				
Original plan	95.23 ± 0.36	99.45 ± 0.29	99.93 ± 0.09	0.07 ± 0.02
1.5	95.57 ± 0.67	99.56 ± 0.24	99.96 ± 0.06	0.07 ± 0.02
3	94.29 ± 1.53^#^	99.28 ± 0.39	99.93 ± 0.07	0.08 ± 0.01^#^
4.5	91.95 ± 2.66^#^	98.52 ± 0.85^#^	99.82 ± 0.21^#^	0.10 ± 0.02^#^
6	88.81 ± 3.85^#^	96.76 ± 2.31^#^	99.16 ± 1.16^#^	0.14 ± 0.04^#^
7.5	86.26 ± 3.87^#^	95.53 ± 2.31^#^	98.78 ± 1.32^#^	0.16 ± 0.04^#^
9	83.59 ± 3.96^#^	93.41 ± 3.06^#^	97.72 ± 2.13^#^	0.20 ± 0.06^#^
Decreased couch height (mm)				
1.5	93.26 ± 0.75^#^	98.87 ± 0.53^#^	99.79 ± 0.20^#^	0.08 ± 0.02^#^
3	90.51 ± 1.37^#^	97.66 ± 0.92^#^	99.42 ± 0.49^#^	0.11 ± 0.03^#^
4.5	87.28 ± 1.99^#^	95.73 ± 1.35^#^	98.67 ± 0.92^#^	0.15 ± 0.04^#^
6	84.15 ± 2.43^#^	93.21 ± 1.82^#^	97.45 ± 1.44^#^	0.20 ± 0.09^#^
7.5	81.43 ± 2.92^#^	90.55 ± 2.53^#^	95.9 ± 2.08^#^	0.25 ± 0.09^#^
9	78.53 ± 3.06^#^	87.27 ± 2.67^#^	93.73 ± 2.46^#^	0.32 ± 0.11^#^

#: indicated a difference in couch height compared with the original plan, *p* < 0.05.

### Evaluation of OAR indicator

Compared with the original plan, various evaluation indicators of OARs were increased with the increase of the treatment couch height and decreased with the decrease of the treatment couch height (Fig. 4). Compared with the original plan, when the couch height was increased by 3 mm, the D_mean_, V_30_, and V_20_ of the ipsilateral lung were increased by 35.12%, 16.35%, and 10.6%, respectively (all *p* < 0.05); Decreasing the couch height could protect OAR to a certain extent, in which, when the couch height was decreased by 3 mm, D_mean_, V_30_, and V_20_ of the ipsilateral lung were decreased by 7.9%, 15.79%, and 10.44%, respectively (all *p* < 0.05) (Table 2).

**Table 2:** Effects of changes in couch height on the radiation dose in OARs.

N = 20	Heart				Ipsilateral lung				Contralateral lung	
	D_mean_ (Gy)	V_30_ (%)	V_20_ (%)	V_10_ (%)	D_mean_ (Gy)	V_30_ (%)	V_20_ (%)	V_10_ (%)	D_mean_ (Gy)	V_5_ (%)
Increased couch height (mm)										
NO shift	5.03 ± 1.61	2.78 ± 1.98	5.11 ± 3.23	9.72 ± 5.20	13.08 ± 1.07	16.21 ± 2.17	24.34 ± 2.29	38.54 ± 3.34	1.89 ± 0.24	5.33 ± 2.63
1.5	5.35 ± 1.69	3.31 ± 2.2	6.10 ± 3.70	10.74 ± 5.45	13.62 ± 1.06	17.58 ± 2.23	25.63 ± 2.28	39.62 ± 3.27	1.95 ± 0.25	6.13 ± 2.85
3	5.71 ± 1.77	3.92 ± 2.44	6.61 ± 3.72	11.85 ± 5.67	14.16 ± 1.06^#^	18.86 ± 2.19^#^	26.92 ± 2.27^#^	40.68 ± 3.20	2.02 ± 0.27	6.98 ± 3.07
4.5	6.07 ± 1.82^#^	4.61 ± 2.67^#^	7.50 ± 3.96^#^	13.04 ± 0.88^#^	14.72 ± 1.06^#^	20.2 ± 2.21^#^	28.2 ± 2.27^#^	41.78 ± 3.14^#^	2.09 ± 0.29^#^	7.88 ± 3.28^#^
6	6.12 ± 1.96^#^	4.67 ± 2.66^#^	7.54 ± 3.89^#^	13.15 ± 6.04^#^	14.68 ± 1.96^#^	19.99 ± 4.27^#^	27.97 ± 4.23^#^	41.45 ± 4.76^#^	2.18 ± 0.32^#^	8.05 ± 3.95^#^
7.5	6.99 ± 1.97^#^	6.21 ± 3.12^#^	9.57 ± 4.40^#^	15.70 ± 6.20^#^	15.87 ± 1.06^#^	22.95 ± 2.25^#^	30.78 ± 2.3^#^	43.83 ± 3.05^#^	2.27 ± 0.34^#^	9.81 ± 3.65^#^
9	7.51 ± 2.03^#^	7.18 ± 3.33^#^	10.71 ± 4.58^#^	17.16 ± 6.30^#^	16.55 ± 1.09^#^	24.36 ± 2.29^#^	32.08 ± 2.33^#^	44.90 ± 3.03^#^	2.36 ± 0.37^#^	10.87 ± 3.80^#^
Decreased couch height (mm)										
1.5	4.74 ± 1.54	2.32 ± 1.75	4.45 ± 2.97	8.77 ± 4.94	12.55 ± 1.08	14.92 ± 2.14	23.07 ± 2.3	37.48 ± 3.43	1.83 ± 0.23	4.64 ± 2.39
3	4.49 ± 1.46	1.92 ± 1.54	3.86 ± 2.71	7.91 ± 4.67	12.05 ± 1.08^#^	13.65 ± 2.12^#^	21.8 ± 2.31^#^	36.41 ± 3.54	1.79 ± 0.23	4.03 ± 2.18
4.5	4.26 ± 1.39	1.58 ± 1.35	3.34 ± 2.46	7.12 ± 4.39	11.56 ± 1.09^#^	12.41 ± 2.08^#^	20.54 ± 2.32^#^	35.34 ± 3.64^#^	1.74 ± 0.23	3.53 ± 2.01^#^
6	4.05 ± 1.32	1.29 ± 1.17	2.85 ± 2.20	6.40 ± 4.11^#^	11.11 ± 1.10^#^	11.22 ± 2.03^#^	19.31 ± 2.32^#^	34.30 ± 3.76^#^	1.70 ± 0.24^#^	3.43 ± 2.29^#^
7.5	3.89 ± 1.26^#^	1.07 ± 1.00	2.48 ± 1.96	5.82 ± 3.84^#^	10.71 ± 1.09^#^	10.18 ± 2.07^#^	18.23 ± 2.38^#^	33.40 ± 3.87^#^	1.68 ± 0.25^#^	2.81 ± 1.81^#^
9	3.67 ± 1.17^#^	0.86 ± 0.87^#^	2.07 ± 1.75^#^	5.17 ± 3.55^#^	10.27 ± 1.09^#^	9.00 ± 1.88^#^	16.95 ± 2.27^#^	32.33 ± 3.98^#^	1.66 ± 0.26^#^	2.57 ± 1.77^#^

#: indicated a difference in couch height compared with the original plan, *p* < 0.05.

**Fig. 4:**
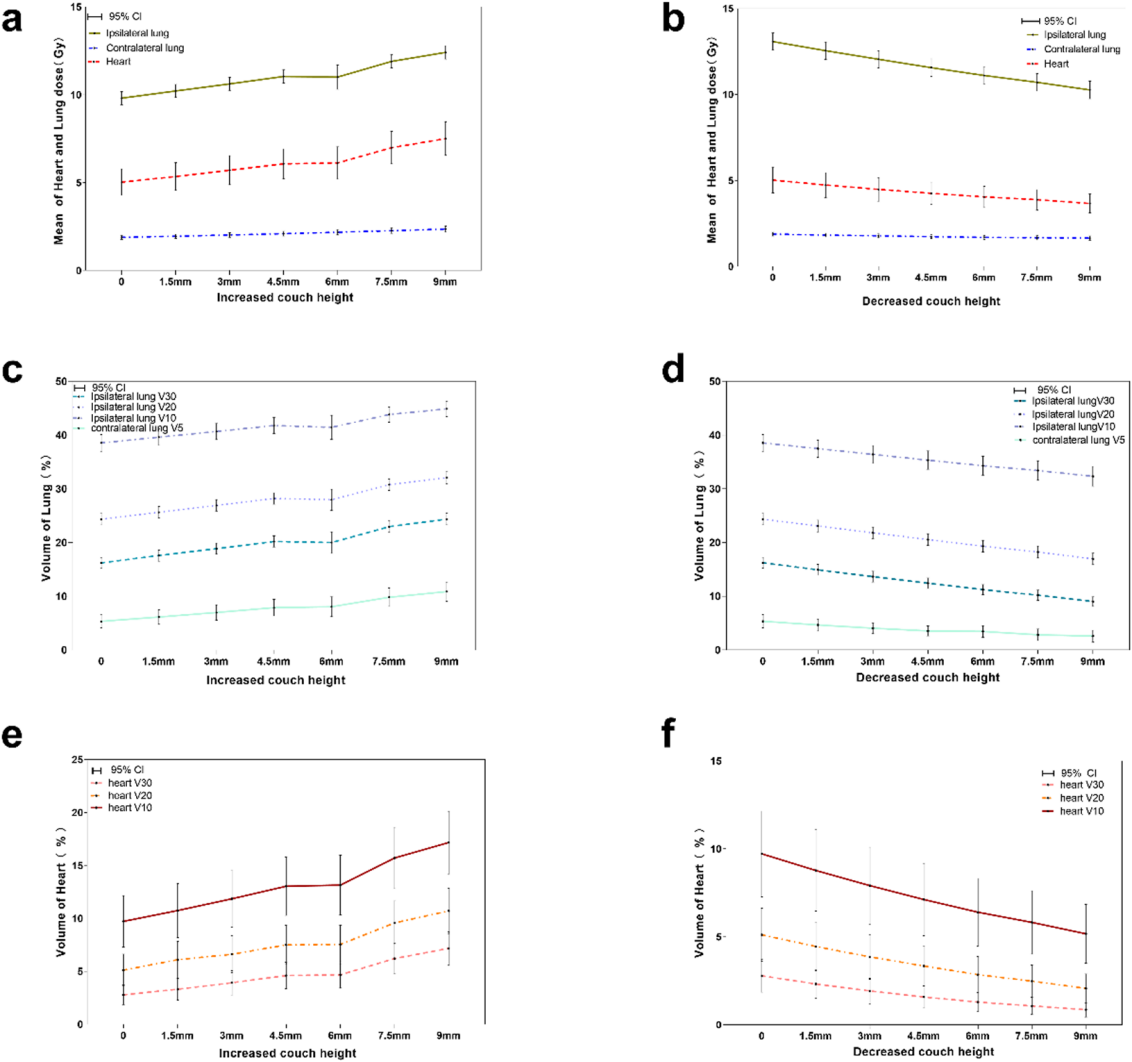
Effect of couch height changes on the radiation dose distribution in OARs. (**a**, **b**) Effect of increasing and decreasing couch heights on mean irradiated doses in heart and lungs. (**c**, **d**) Effects of increasing and decreasing couch heights on irradiated volume in lungs. (**e**, **f**) Effects of increasing and decreasing couch heights on the irradiated volume in heart.

### Comparative analysis of positioning errors in the ventral-dorsal directions

**Fig. 5:**
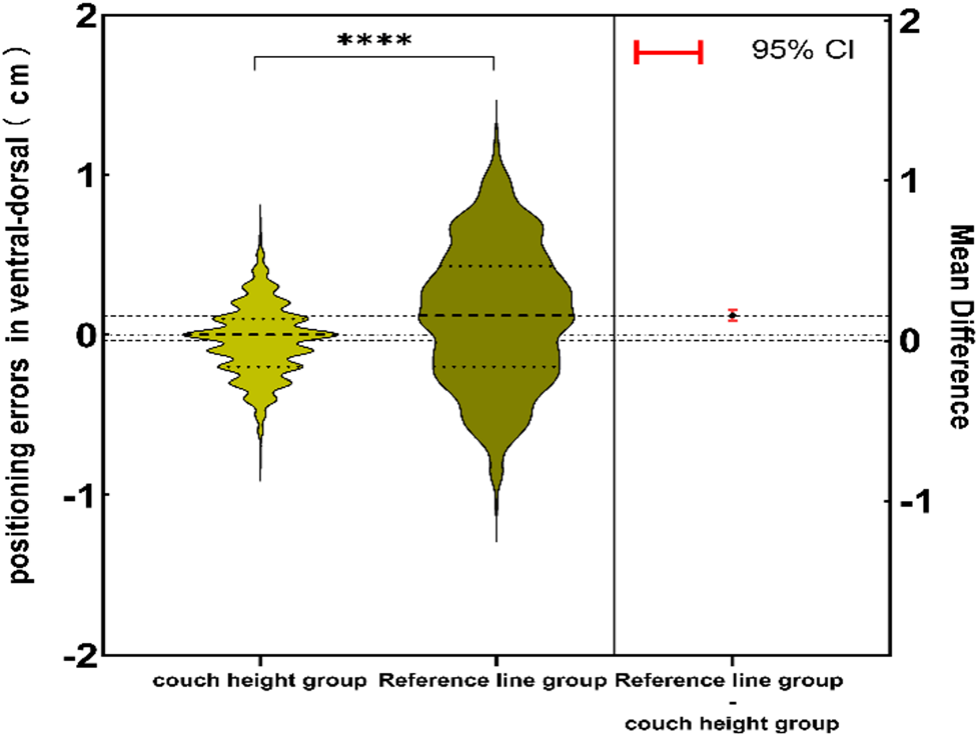
Diagram of estimated positioning errors between the couch height group and the reference line group.

**Fig. 6:**
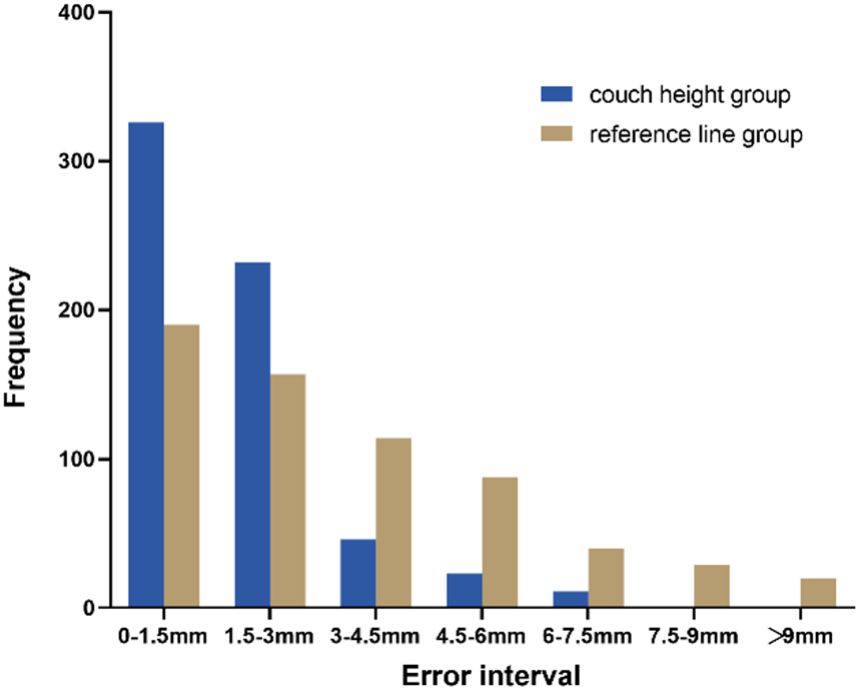
The distribution of absolute positioning error.

The comparison of absolute positioning error showed that the positioning errors in the ventral-dorsal directions were (0.17 ± 0.15 cm) in the couch height group and (0.36 ± 0.25 cm) in the reference line group (*p* < 0.05) (Fig. 5); the positioning referring to the treatment couch height reduced the absolute positioning errors in the ventral-dorsal directions by 52.4%; the percentages of the absolute positioning errors within 1.5 mm, 3 mm and 4.5 mm were 52.4%, 88.7% and 94.7% respectively in the couch height group and 29.8%, 54.4% and 86.1% respectively in the reference line group (all *p* < 0.05) (Fig. 6). Based on the analysis of the effects of positioning errors on the doses in PTV and OARs, it was found that a 3 mm positioning error significantly affected the doses in PTV and OARs (*p* < 0.05). Therefore, we consider positioning errors greater than 3 mm as poor and those equal to or less than 3 mm as perfect. The positioning error pass rate in the reference couch height group is significantly better than that in the reference line group (Table 3).

**Table 3:** Different positioning States with different groups.

		Positioning state (%)		
		≤ 3 mm	>3 mm	Total
Group	Couch height group	564(88.4)	74(11.6)	638
	Reference line group	311(48.7)	327(51.3)	638
Total		875(68.6)	401(31.4)	1276
χ2		232.8 < 0.001		
p				

### Consistency of the couch height-based positioning

**Fig. 7:**
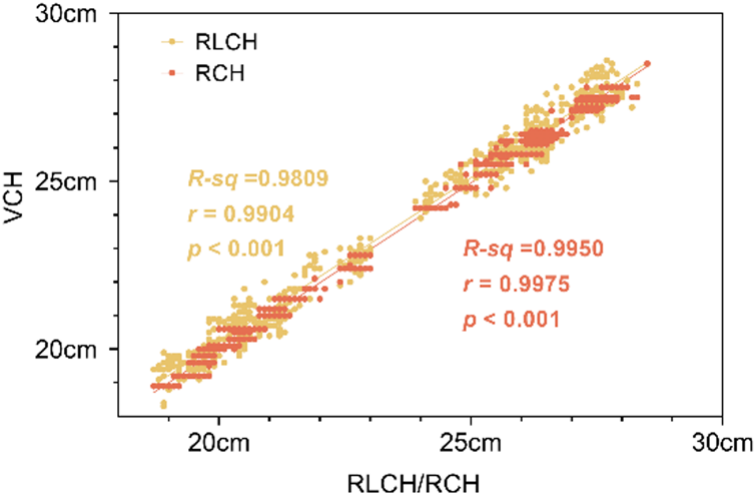
Pearson’s correlation analysis of couch heights in three groups. RCH: Reference couch height; RLCH: Reference line-based couch height, VCH: Verified couch height.

Based on the analysis of data on couch heights, a strong correlation between RCH and VCH was observed, and the coefficient of determination (R^2^ = 0.9950) and Pearson correlation coefficient (*r* = 0.9975) between RCH and VCH were significantly higher than those (R^2^ = 0.9809, *r* = 0.9904) between the RLCH and VCH, with a statistical significance of *P* < 0.001 (Fig. 7). The above results indicated a markedly stronger correlation and superior consistency for the couch height-based positioning compared to the reference line-based positioning.

### Calculation of MPTV

The reference line group exhibited larger positioning errors (0.36 ± 0.25 cm), with a MPTV of 1.08 cm in the ventral-dorsal direction, while the couch height group demonstrated significantly reduced positioning errors (0.17 ± 0.15 cm, *p* < 0.05), with a MPTV of 0.53 cm in the ventral-dorsal direction, which was decreased by 51% compared with the reference line group. This substantial decrease in the necessary MPTV was consistent with the significantly higher percentage of positioning errors within 3 mm in couch height group compared with the reference line group (88.7% vs. 54.4%, *p* < 0.05). These results quantitatively demonstrated that adopting the couch height-based positioning method allows for a markedly smaller MPTV without compromising target coverage, thereby potentially reducing radiation exposure to adjacent OARs.

## Discussion

Radiation therapy is a double-edged sword; it can effectively destroy tumors but may also damage normal tissues such as the heart and lungs, leading to side effects such as radiation pneumonitis and cardiac complications^21^, which can severely affect the quality of life and even increase the potential risk of secondary malignancies in patients^22^. Therefore, ensuring treatment precision and minimizing positioning errors are crucial for maximizing the benefits of radiotherapy.

This study focuses on postoperative radiotherapy for left-sided breast cancer, systematically evaluating the effects of ventral-dorsal positioning errors on radiation dose distribution in PTV and radiation dose exposure in OARs. According to the study by Darby et al.^23^, an increase of 1 Gy in mean heart dose (MHD) is associated with an increase of 7.4% risk in major coronary events. This implies that excessive cardiac irradiation caused by positioning errors may increase the risk of long-term death from heart disease in patients, thereby reducing the overall survival rate. Furthermore, inadequate dose in target volume may also increase the risk of local recurrence in high-risk breast cancer patients after radical mastectomy, further affecting the overall survival rate^24^. In this study, dorsal positioning errors of 3–9 mm increased MHD by 0.68–2.48 Gy, corresponding to a 5-18.4% increase in late cardiac toxicity risk. Meanwhile, a ventral positioning error of 3–9 mm reduced the PTV V50 by 4.96–17.54%, leading to a radiation loss of approximately 2.4–8.7 Gy (prescription dose 50 Gy) within the PTV, thus increasing the risk of local recurrence. Compared with 51.3% of positioning errors > 3 mm in the reference line group, the couch height group exhibited 12.5% positioning errors > 3 mm, highlighting its advantage in ensuring adequate coverage of the target volume while effectively protecting the heart. In addition, V_30_ > 10% in lung is significantly correlated with the incidence of symptomatic radiation pneumonitis^25^. Calculated using the van Herk formula^20^, the required MPTV margin reduced to 5.3 mm in the couch height group, which reduced by 51% compared with 10.8 mm in the reference line group. This means that with more precise positioning, the irradiation volume can be significantly reduced while ensuring adequate target coverage, thereby better protecting surrounding normal tissues. The couch height-based positioning can be used to help control V_30_ in lung below the safety threshold. We cautiously note that other intra- and inter-fraction variable factors, such as tumor shrinkage and organ motion, must also be comprehensively considered. More in-depth research must be conducted before the use of smaller margins in clinical practice.

Compared with a previous study^6^, in which positioning errors were simulated at 2.5 mm intervals with a maximum error of 15 mm. In this, finer simulation intervals (1.5 mm, maximum 9 mm) were used, and the positioning errors in both ventral and dorsal directions were investigated. In particular, the effects of ventral-dorsal positioning errors on the dose distribution within the PTV and the irradiated dose in OARs were evaluated respectively, thereby providing more accurate data on dosimetric changes.

This study showed that when a dorsal positioning error of 3 mm occurred, the D_mean_, V_30_, and V_20_ in the ipsilateral lung were increased by 35.12%, 16.35%, and 10.6%, respectively. Even a ventral positioning error of 1.5 mm would cause a 2.07% decrease in V_50_ in PTV. These findings are consistent with those of Liao et al.^26^, who evaluated the dosimetric effects on CTV in chest wall (CTV_Cw_) and CTV in supraclavicular region (CTVsc), respectively, and the results showed that a ventral positioning error of 3 mm reduces D_95_ and D_98_ in CTVcw by 1.4% and 2.8%, respectively, and in CTVsc by 0.7% and 1.4%, respectively. On the other hand, the dorsal positioning errors primarily result in inclusion of more cardiac and pulmonary tissues into the radiation field, thereby leading to an increased irradiated dose in OARs: a 3 mm dorsal positioning error increased V_25_ in heart by 1.6% and V_20_ in the ipsilateral lung by 2.4%. These findings consistently demonstrate that ventral-dorsal positioning errors not only significantly compromise the target coverage, but also markedly increase irradiated dose in adjacent normal tissues. Therefore, ventral-dorsal positioning errors should be paid great attention to and strictly controlled in clinical practice.

Against this backdrop, the couch height-based positioning method demonstrates significant advantages. The findings of this study revealed that 88.7% of ventral-dorsal errors in the couch height group were controlled within 3 mm, indicating superior error-control capabilities compared with the reference line group (54.4%, *p* < 0.05). This finding is consistent with that of Greer et al.^11^, which indicated that couch height–based positioning for pelvic radiotherapy reduces systematic error (Σ) from 3.7 mm to 1.2 mm, random error (σ) from 2.3 mm to 1.3 mm, and total error from 4.6 mm to 1.7 mm. Similarly, Ohira et al.^12^ reported that couch height-based positioning for abdominal radiotherapy reduces Σ from 2.6 mm to 0.8 mm and σ from 2.1 mm to 0.9 mm, thereby decreasing the MPTV from 6.7 mm to 2.2 mm. Petillion et al.^27^ applied this technique in whole-breast radiotherapy, and the results showed reductions of Σ from 5.7 mm to 1.8 mm and σ from 4.6 mm to 2.2 mm, with significantly lowered MPTV. However, the findings of Petillion et al.^27^ were based on offline verification, whereas the findings of this study were based on online verification, which not only quantified the magnitude of positioning errors between the two methods but also confirmed the correlations among RCH, RLCH, and VCH. Notably, the correlation between RCH and VCH (*r* = 0.9975) was superior to that between RLCH and VCH (*r* = 0.9904), further substantiating the real-time high accuracy of couch height-based positioning.

While SGRT has been shown to improve translational positioning accuracy in breast radiotherapy the evaluation of rotational errors remains limited^28^. Lai et al.^7^ compared SGRT, skin marking line-based positioning with a combined method in DIBH breast cancer radiotherapy, demonstrating that the combined method results in superior control of translational errors (70% within 3 mm) and rotational errors (over 37% within 1°), while also improving the positioning efficiency. This strategy is therefore considered an optimal method for DIBH breast radiotherapy and may also be applicable under free-breathing conditions. Nonetheless, the widespread use of SGRT is restricted by high cost and time demands, thus limiting its adoption in high patient volumes or resource-constrained centers. In this context, this study introduces couch height-based positioning, which achieved ventral–dorsal positioning accuracy comparable to SGRT and superior to skin marking line-based positioning, without the need for additional equipment. By leveraging the stable geometric relationship between the skeleton and the treatment couch, this approach provides a practical and cost-effective alternative. However, the current findings apply only to left-sided breast cancers, and further validation in right-sided breast cancers is warranted.

The comparison of absolute positioning error between two groups revealed that the couch height group demonstrated a statistically significant improvement in positioning accuracy compared with the reference line group, which was further substantiated by a post-hoc power analysis using a power analysis software program (PASS 2021; NCSS, LLC) based on the empirical data obtained in our study (mean difference = 0.20, standard deviations [SD] of differences = 0.26), a sample size of 60 and a significance level of α = 0.05 confirmed a statistical power exceeding 99.9% for detecting the inter-group difference. The large effect size (Cohen’s d = 0.77) indicated that this study was amply powered to identify a clinically significant difference.

### Limitations

This study also has some limitations. Notably, due to equipment constraints, the potential influence of respiratory motion such as with DIBH, or the use of advanced rigid immobilization systems was not investigated. Furthermore, verification based on long-term clinical endpoints, including overall survival and cardiac event incidence, is still lacking. Future studies should prioritize multicenter, large-sample prospective trials to optimize the integration of the couch height-based positioning technique with volumetric modulated arc therapy (VMAT). Meanwhile, dose distribution in the chest wall and regional lymph nodes should be assessed respectively, and the synergistic effects of the above-mentioned integrated strategies should be explored to not only reduce ventral-dorsal positioning errors but also mitigate respiratory-induced uncertainties, thereby achieving enhanced cardiac protection. Ultimately, future studies should aim to ultimately validating the positive impacts of integrated strategies on patients’ long-term qualities of life.

## Conclusions

The couch height-based positioning is an effective, reliable, and easily implemented strategy, which significantly improves ventral-dorsal positioning accuracy during radiotherapy for left-sided breast cancer, enhances dose distribution, reduces the exposure dose in OARs, and has an important clinical practical value and excellent promotion prospects.

## Data Availability

The datasets generated during the current study are not publicly available due to patients’ privacy reasons but are available from the corresponding author on reasonable request.
